# Extracting structured data from organic synthesis procedures using a fine-tuned large language model†

**DOI:** 10.1039/d4dd00091a

**Published:** 2024-07-31

**Authors:** Qianxiang Ai, Fanwang Meng, Jiale Shi, Brenden Pelkie, Connor W. Coley

**Affiliations:** a Department of Chemical Engineering, Massachusetts Institute of Technology Cambridge MA USA ccoley@mit.edu; b Department of Chemical Engineering, University of Washington Seattle WA USA

## Abstract

The popularity of data-driven approaches and machine learning (ML) techniques in the field of organic chemistry and its various subfields has increased the value of structured reaction data. Most data in chemistry is represented by unstructured text, and despite the vastness of the organic chemistry literature (papers, patents), manual conversion from unstructured text to structured data remains a largely manual endeavor. Software tools for this task would facilitate downstream applications such as reaction prediction and condition recommendation. In this study, we fine-tune a large language model (LLM) to extract reaction information from organic synthesis procedure text into structured data following the Open Reaction Database (ORD) schema, a comprehensive data structure designed for organic reactions. The fine-tuned model produces syntactically correct ORD records with an average accuracy of 91.25% for ORD “messages” (*e.g.*, full compound, workups, or condition definitions) and 92.25% for individual data fields (*e.g.*, compound identifiers, mass quantities), with the ability to recognize compound-referencing tokens and to infer reaction roles. We investigate its failure modes and evaluate performance on specific subtasks such as reaction role classification.

## Abstract 2

An open-source fine-tuned large language model can extract reaction information from organic synthesis procedure text into structured data that follows the Open Reaction Database (ORD) schema.

## Graphical abstract

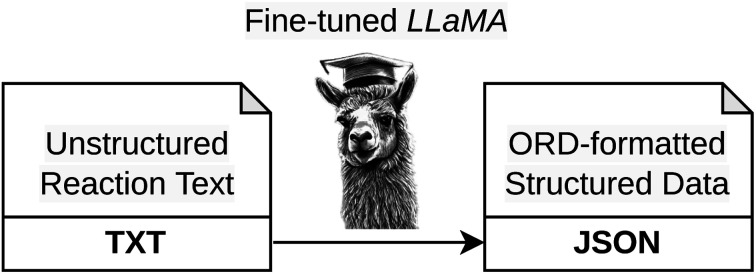

## 1 Introduction

Data-driven methods are now routinely employed in the physical sciences. A trend toward the use of supervised machine learning (ML) techniques has increased the need for structured data, *i.e.*, data represented using a standardized data schema. In most scientific communities, however, data is stored and communicated predominantly *via* unstructured documents and prose, with only a few exceptions.^1^ Synthetic organic chemistry is not one of those exceptions. Reaction procedures and details are commonly recorded as free text in journal publications, patents, or electronic lab notebooks (ELNs). Manual information extraction and curation are still widely used to construct structured datasets from unstructured texts.^2,3^ An automated method to extract structured reaction data from unstructured texts would accelerate efforts to use historical reaction data for data-driven discovery.

As an information extraction task, structured data extraction from text can be considered as a combination of named entity recognition (NER) and relation extraction (RE) between named entities. Challenges in chemical NER include the pervasive usage of abbreviations and aliases, deviations from standard nomenclature, and the ambiguous boundaries between which a chemical entity is defined (*e.g.*, when multiple words describe a single species).^4,5^ A variety of methods have been applied for chemical NER tasks. Rule-based or dictionary-based methods, such as LeadMine^6^ and ChemicalTagger,^7^ have been used to annotate reaction procedure texts or in the text parsing pipeline for constructing synthesis datasets such as SureCHEMBL,^8^ Pistachio,^9^ and ZeoSyn.^10^ While these algorithms are usually computationally efficient, the scope of rules and dictionary items limits their generalizability to new datasets. Various statistical model-based NER algorithms have also been proposed, often as a sequence labeling problem where the tokens in a sentence are assigned most likely tags based on token features. A popular strategy is the use of conditional random fields^11^ in combination with expert-selected features^12^ or contextualized word embeddings from neural networks (recurrent networks,^13–15^ or transformers^16–19^).

Traditionally, RE is formulated as a downstream task to NER and is solved as an ensemble of classification problems for entity pairs.^20,21^ More recent efforts aim to solve NER and RE simultaneously by building end-to-end models.^22–25^ This trend has persisted as pretrained large language models (LLMs) have become more accessible. LLMs have been used for NER/RE tasks in biomedicine,^26^ materials,^27^ and clinical trials,^28^ showing promise as tools for structured data extraction. For example, Dagdelen *et al.* developed a training pipeline for GPT-3 to extract information from scientific texts about crystalline materials as structured JSON^29^ and Walker *et al.* present an iterative scheme to fine-tune LLMs for extracting structured data of gold nanorods synthesis.^30^ Recent studies by Zhong *et al.* explored fine-tuned LLMs for reaction data extraction from literature in PDF format.^31,32^ The output of these models provides a reasonable coverage of reaction information, with the exception of quantity information. Pretrained LLMs can also be used for this task directly without fine-tuning. For example, a recent preprint by Patiny and Godin explores extracting analytical experiment results from literature solely through prompt engineering.^33^ While this method can extract structured data by including in-prompt data schema, it relies on closed-source LLMs and performs poorly when numerical values are involved.

One important use case for extracting structured reaction data is the production of procedural instructions to be used for reproducing experiments. For example, Vaucher *et al.* developed a transformer-based model to translate sentences of experiment procedures into action sequences.^34^ While these action sequences contain detailed information for execution, their evaluations focus more on the type of action than the parameters or objects of that action. SynthReader,^35^ a rule-based translator developed by Mehr *et al.*,^35^ converts natural language procedures to χDL, a data schema designed for chemical operations. Such a rule-based method, despite being computationally efficient, has to be expanded/modified to adapt to a different distribution, *e.g.*, a change in writing style. Various submissions to Cheminformatics Elsevier Melbourne University (ChEMU) evaluation lab^36–38^ also aim to solve the NER/RE tasks including reaction/workup steps. Since these campaigns aim at evaluating individual NER/RE tasks, they do not constitute an end-to-end solution for structured data extraction into a specific output data schema.

**Fig. 1:**
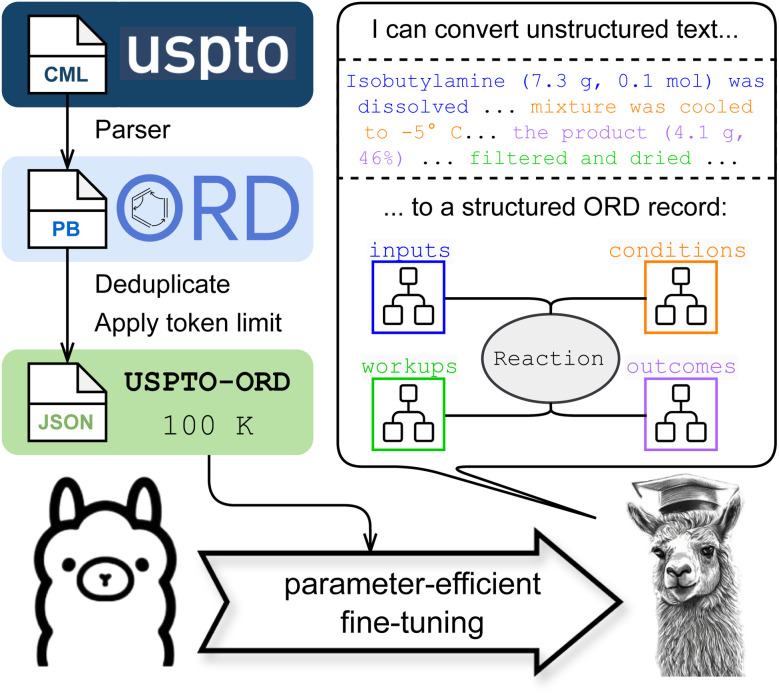
Overview of this study's approach to structured reaction data extraction from text. A 100k reaction subset of the United States Patent and Trademark Office (USPTO) reaction data^39^ as represented in the Open Reaction Database (ORD)^40^ is used to fine-tune and evaluate LLaMa-2-7B. An example of the structured ORD record is included in Section 2.1. The data pipeline (top left) is detailed in Section 2.2. The fine-tuning procedure is described in Section 2.3. The llama with a cap was generated using Craiyon AI.^41^

In this study, we fine-tune an open-source large language model to extract structured reaction information from unstructured text from US patents (Fig. 1). To structure the desired outputs, we adopt the Open Reaction Database (ORD) data format, a comprehensive data schema tailored to organic reactions.^40^ The 100 000-reaction dataset we use for fine-tuning is part of a collection originally published by Lowe in Chemical Markup Language (CML) format,^39^ so the fine-tuned model essentially pursues the same goal as Lowe's expert natural language processing pipeline, albeit using a different data schema. Extracted records cover information on reactants, products, conditions, and workup steps. We demonstrate that the fine-tuned model produces syntactically correct ORD records from the USPTO with an average accuracy of 91.25% for chemical messages (compounds, workups, conditions) and 92.25% for individual data fields. We also investigate its failure modes and evaluate performance on reaction role classification. We note that a preliminary version of this study was previously disclosed as part of a Perspective article on opportunities for LLMs in chemistry.^42^

## 2 Methods

### 2.1 Introduction to the Open Reaction Database (ORD) schema

**Fig. 2:**
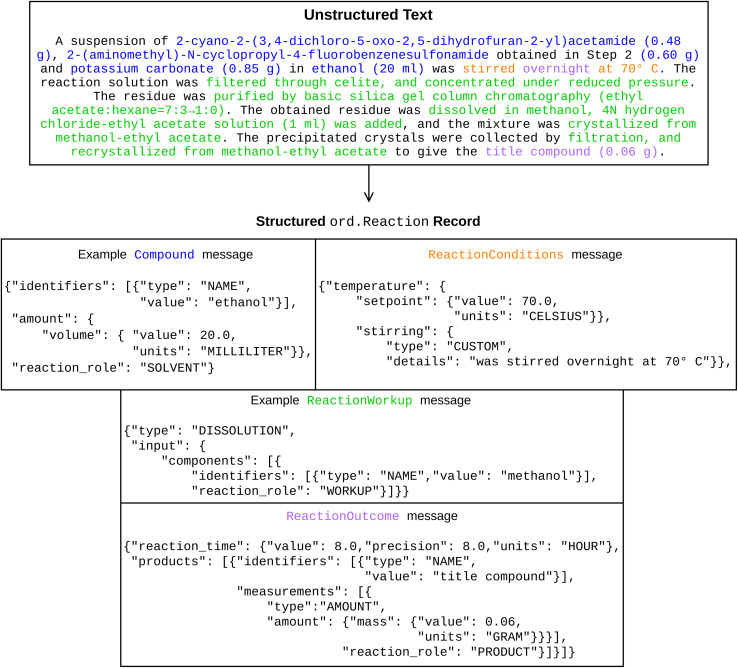
(Top) The original text description of a reaction procedure and (bottom) example messages within the structured ORD reaction record.^43^

A reaction record in the ORD is structured as a Reaction message using Google's Protocol Buffers, which can be faithfully converted to and from JSON format without loss of information. For a specific Reaction, we focus on four chemically important fields: inputs, conditions, workups, and outcomes, each of which is also a message or a list of messages defined in ORD schema. An example reaction record is shown in Fig. 2 with representative fields populated. There are more than 600 fields defined in ORD schema, some of which are size-mutable, and an ORD record typically includes many nested messages. There are also strict rules on types and values admitted by data fields. For example, the type field of ReactionWorkup is an enum field that only accepts specific strings, and assigning out-of-vocabulary strings to this field leads to a syntactically invalid ORD record. The full definition of the Reaction message used in this study is available on GitHub.^44^

### 2.2 Dataset preparation from patents and the ORD

Reaction records from the United States Patent and Trademark Office (USPTO) were collected from the ORD, sharded across 489 datasets. The link to a complete list of dataset IDs can be found in the ESI.† These records were originally published by Lowe in Chemical Markup Language (CML) format^39^ and were imported into the ORD using a custom CML-to-ORD translation script.^45^ A reaction record is admitted to our dataset if it satisfies the following conditions:

• Each of its ReactionInput messages has non-empty values for its components field. This usually means this reaction input is not the crude product of another reaction and that the chemical information of this reaction's inputs are present in reaction procedure text.

• The reaction includes an associated procedure text, *i.e.*, the notes.procedure_details field of this reaction is a paragraph describing the reaction.

Reaction records satisfying these criteria were exported to JSON and deduplicated using OpenAI's data preparation tools (openai tools fine_tunes.prepare_data) to produce 1 339 260 unique records. The use of OpenAI's data preparation tools is free and was used here solely for convenient prompt deduplication. The procedure text and structured JSON are combined using a prompt template (see ESI†) modified from Stanford Alpaca.^46^ A sequence length limit of 2048 tokens based on LLaMA tokenizer, is imposed due to memory considerations in fine-tuning the language models. This sequence limit reduces the number of records to 1 300 613 (97.1%) of 1 339 260. The cumulative distribution function of sequence lengths is shown in Fig. S1.† A subset of 100K records, hereinafter referred to as USPTO-ORD-100K, is randomly selected from the 1 300 613 records. Unless otherwise specified, a random 8 : 1 : 1 train : validation : test split is applied to USPTO-ORD-100K to train/evaluate models throughout this study. This data pipeline is schematically shown in Fig. 1.

The information in a structured ORD record is not guaranteed to be a proper subset of its free text description, as some information in the structured ORD record is derived from elsewhere, and in this work denoted “implicit information”. For example, the reaction roles of compounds are rarely stated in a reaction's text description. As another example, the text description may indicate a filtration step (mapping to a ReactionWorkup of type FILTRATION in its ORD record) but does not include “filter” or “filtration” explicitly, *e.g.*, “passing through celite”. We consider this kind of implicit information learnable and therefore do not exclude them from ORD records. On the other hand, some implicit information is considered unlearnable and thus excluded from the ORD records. Specifically,

• Unspecified outcome: if the name of a product is present in the ORD record and is not explicitly stated in the reaction text, this name is removed from the ORD record. This could happen when the product name is defined only in the title of the corresponding patent and not mentioned explicitly in the procedure text. This can also happen for reactants when they are referred to by compound identifiers or generic names.

• Calculated yield: if the yield value of a product is present in the ORD record and its integer value is not explicitly stated in the reaction text, this value is removed from the ORD record. This can occur when the calculated yield is different from the yield reported in the procedure text.

### 2.3 LLaMA fine-tuning procedure

LLaMA is a collection of decoder-only models first released in February 2023 by Meta AI,^47^ with an updated version LLaMA-2 (released in July 2023).^48^ LLaMA models are convenient foundational models for scientific communities because they are pre-trained using publicly available data only, have parameter sizes ranging from 7 billion to 70 billion, and are distributed with both model weights and training code under an open-source license. We select LLaMA-2-7B in this study for fine-tuning due to memory considerations. We note the pretrain-finetune paradigm is not exclusive to LLaMA nor the decoder-only models, and other large language models are also amenable to this task. Further performance improvements are likely possible by adopting a different pretrained model.

To avoid tuning the entire 7 billion parameters in LLaMA-2-7B, we adopt LLaMA-Adapter in our fine-tuning procedure.^49^ LLaMA-Adapter achieves parameter-efficient fine-tuning using learnable adaption prompts: for each of the topmost *L* transformer layers, a learnable prompt of length *K* is prepended to the (embedded) word tokens. This procedure reduces the total number of trainable parameters to *K* × *L* × *C*, where *C* is the token embedding dimensions, set to 4096 by default in LLaMA. Throughout this study, *K* = 10 and *L* = 30, giving 1.2 million trainable parameters that can fit in a GPU of 24 GB memory in half precision.

The train and validation datasets from the aforementioned random split are used for fine-tuning LLaMA-2-7B. The validation set is used to monitor the training process and to determine the number of training epochs with early stopping. Fine-tuning LLaMA-2-7B for 15 epochs with an initial learning rate of 7 × 10^−5^ was completed in approximately 70 hours using 2 NVIDIA RTX 4090 GPUs. In contrast, preparing the ORD datasets (in .pb.gz format) to obtain USPTO-ORD-100K took approximately 4 hours using our scripts with a 16-core 4.70 GHz CPU (Intel® i7-1260P). The average inference speed was roughly 37 token per second as estimated over 100 generations on one RTX 4090 GPU with batch size set to 1. This model is referred to as “the fine-tuned model” throughout this study. The hyperparameters for fine-tuning were not optimized.

### 2.4 Evaluation protocol and metrics

Text descriptions of reaction records from the test set of USPTO-ORD-100K are passed to the fine-tuned LLaMA-2-7B to generate structured data as text completions for model evaluation. Because a Reaction message consists of nested sub-messages (or “objects” in JSON terminology), such as Compound and ReactionWorkup, we can define evaluation tasks based on the comparison between the ground truth and LLM-inferred Reaction at the message level: Evaluation Metric 1. For a given message type, how many messages of this message type are accurately extracted or erroneously added, removed, or altered?

**Fig. 3:**
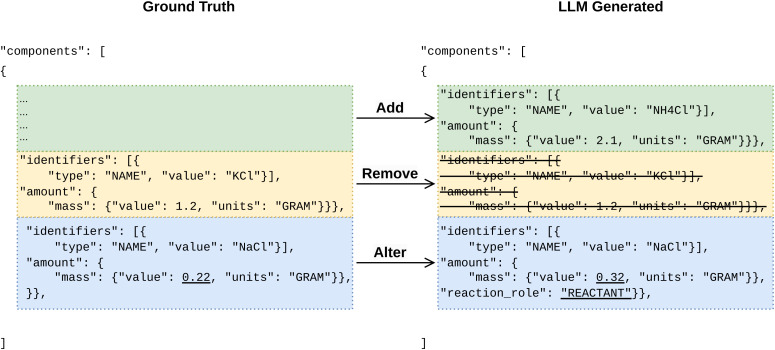
An example of Evaluation Metric 1 for when comparing two lists of Compound messages, where the ground truth denotes the already structured JSON from the test set of USPTO-ORD-100K. Three failure modes at the Compound message level, “Addition”, “Removal”, and “Alteration” are colored green, yellow, and blue, respectively. Underscored fields denote failures at the leaf fields level (Evaluation Metric 2, *vide infra*). Data shown is for illustration purposes only.

Fig. 3 shows an example of Evaluation Metric 1 when comparing two ReactionInput messages given the message type of Compound messages. To distinguish the three failure modes, we first define a distance function for the given message type based on DeepDistance,^50^ an edit distance similar to Levenshtein distance designed for nested objects. When comparing two lists of messages (the shorter list is padded with empty messages such that two lists are of equal sizes), a bijective mapping between messages from two lists is found by minimizing the distance sum of all pairs, which is then used to identify the aforementioned failure modes.

Since a message always has a tree structure, we can also define evaluation tasks at the leaf level, where a leaf corresponds to an unstructured, literal field: Evaluation Metric 2. For a given message type, how many leaf fields of messages of this message type are accurately extracted or erroneously added, removed, or altered?

We note that Evaluation Metric 1 is defined at a lower granularity and is more stringent than Evaluation Metric 2, as summarized in Table 1. For example, in the case shown in Fig. 3, an entire compound message (blue) is marked as altered, while only two leaf fields (underscored) are considered as “Alteration” (value), and “Addition” (reaction_role), respectively. Assigning “Addition” and “Removal” to leaf fields also depends on the assignment at the message level, for example, when a message is assigned “Removal”, all of its leaf fields are assigned “Removal”.

**Table 1:** Comparison between Evaluation Metric 1 and 2

Metric	Specific to a message type	Specific to a field type	What is being counted?	Granularity
1	Yes	No	Added/removed/altered messages	Low
2	Yes	Yes	Added/removed/altered leaf fields	High

It could be reasonable to use a numerical error measure to evaluate field-level extraction. This is because for certain downstream tasks, such as reaction condition recommendation, one could argue that mis-extracted fields containing floating point numbers will have a less deleterious effect on performance if they are close to the true value. However, we prefer the strict evaluation of exact-match accuracy for the information extraction task used here as sometimes missing or misplacing a number can happen more frequently than extracting a wrong number. This is reflected in an analysis on extracting reaction temperature values (ESI Section S7†).

## 3 Results and discussion

### 3.1 Quantitative model evaluation

The fine-tuned LLaMA-2-7B model is evaluated against the test set from the random 8 : 1 : 1 train–validation–test split of USPTO-ORD-100K. Out of the 10K model outputs (completions), only 42 (0.4%) of them are invalid JSON records, and 59 (0.6%) of them are invalid ORD records. Note the former is a sufficient condition for the latter. All of the 42 syntactically JSON invalid completions can be “repaired” by heuristic string operations, such as adding missing quotes or commas, using jsonrepair.^51^ After repairing, 9963 (99.6%) valid ORD records are collected. These results indicate that the fine-tuned model successfully learns the syntax of the ORD's structured data schema during training.

Table 2 summarizes the evaluation results at the message level (Evaluation Metric 1). The fine-tuned model is able to extract compound information for ReactionInput entries reliably with an accuracy of 85.6%. Compared with missing compound information in ReactionInput (5.0%, failure mode “Removal”), it is relatively rare (2.3%) for the model to include excess compounds (failure mode “Addition”), and almost all of the excess compounds come from misplacement (*e.g.*, a ProductCompound is placed in ReactionInput) instead of hallucination.

**Table 2:** Evaluation results at the message level (Evaluation Metric 1) for structured records extracted using the fine-tuned LLaMA-2-7B model. For each record in the test set of USPTO-ORD-100K, an ORD-formatted JSON record is extracted from the unstructured text and evaluated against the ground truth using Evaluation Metric 1. The “Path” column denotes the root path of the corresponding messages in a reaction message. * These values were calculated using a more lenient routine detailed in the main text

Message type	Path	Accurate	Removal	Addition	Alteration	Total
Compound	Inputs	38 470 (85.6%)	2242 (5.0%)	1015 (2.3%)	4242 (9.4%)	44 954
		41 138* (91.5%)			1574* (3.5%)	
ProductCompound	Outcomes	7450 (71.3%)	345 (3.3%)	58 (0.6%)	2656 (25.4%)	10 451
		9105* (87.1%)			1001* (9.6%)	
ReactionConditions	Conditions	9524 (95.7%)	N/A	N/A	433 (4.4%)	9957
ReactionWorkup	Workups	44 165 (90.7%)	1713 (3.5%)	1719 (3.5%)	2807 (5.8%)	48 685

Errors in extracting ProductCompound entries are more frequent, as indicated by a lower accuracy of 71.3%. Upon inspection, we noticed the errors mainly originate from implicit information: some fields of a ProductCompound message are not explicitly stated in the text description and are instead derived or inferred. One example is the “calculated” reaction yield, in contrast to the “reported” reaction yield which the model can capture successfully (Table S2†). To alleviate this effect, we also report the accuracy using a more lenient routine for identifying equivalent ProductCompound messages that considers two ProductCompound messages identical if all of their identifiers and amount fields are identical. These fields often capture all important chemical information about reaction outcomes. After applying this less strict equivalence definition, the accuracy for extracting ProductCompound messages increases from 71.3% to 87.1%, indicating that the model is capable of chemical entity/relation extraction even if it struggles with implicit calculation of yields. This routine also results in an increased accuracy (91.5%) for Compound messages in ReactionInput by excluding errors in reaction role classification (*vide infra*).

High accuracies of 95.7% and 90.7% are measured for ReactionConditions and ReactionWorkup, respectively. Since the ORD schema defines ReactionConditions as one single message rather than a list of messages, no “Addition” or “Removal” of this type of message is applicable.

To further understand how the fine-tuned model performs in extracting different types of chemical information, the completions are examined with finer granularity at the leaf level (Evaluation Metric 2), as shown in Table 3. The fine-tuned model shows excellent recognition capability for chemical entities such as compound identifiers (accuracy 93.5%) and amounts (95.2%), and it can infer reaction roles that are usually not explicitly stated in procedure texts (Section 3.3). Errors at the field-level mainly come from implicit information in ProductCompound messages, such as calculated yields (Table S1†).

**Table 3:** Evaluation results at the leaf field level (Evaluation Metric 2) for structured records extracted using the fine-tuned LLaMA-7B model. For each record in the test set of USPTO-ORD-100K, an ORD-formatted JSON record is extracted from the unstructured text and evaluated against the ground truth using Evaluation Metric 2. * These fields do not belong to any of the five field types (identifiers, amount, reaction role, condition, workup). In this dataset, all of them are leaf fields of ProductCompound, including texture, isolated_color, and yield-related measurements

Message type	Field type	Accurate	Removal	Addition	Alteration	Total
ProductCompound & Compound	Identifiers	100 958 (93.5%)	5490 (5.1%)	2590 (2.4%)	1566 (1.5%)	108 014
	Amount	74 209 (95.2%)	3434 (4.4%)	2182 (2.8%)	300 (0.4%)	77 943
	Reaction role	48 262 (89.3%)	2797 (5.2%)	1264 (2.3%)	2978 (5.5%)	54 037
ReactionConditions	Condition	26 782 (98.3%)	298 (1.1%)	391 (1.4%)	176 (0.7%)	27 256
ReactionWorkup	Workup	178 733 (94.0%)	8360 (4.4%)	10 189 (5.4%)	3156 (1.7%)	190 249
Other*		31 794 (84.80%)	5261 (14.0%)	2240 (6.0%)	439 (1.2%)	37 494

As an alternate approach and point of comparison, we explored extracting structured data with pretrained LLMs directly using the chain-of-thought prompting method,^52^ a few-shot training method by engineering the prompts such that they mimic the thought processes of a human when solving a complicated task. This method is easier to deploy compared to the fine-tuning methods; however, it could only produce syntactically correct ORD data in 408 out of 500 cases after repairing with accuracies of 61.2% and 31.3% for Compound and ProductCompound, respectively, indicating that chain-of-thought prompting without fine-tuning is likely insufficient for this task. This prompting method is also limited by human-crafted instructions and the context window of the model, and, considering there are more than 600 different fields defined in ORD schema, preparing examples and steps to extract a full Reaction record seems impractical. Enabling JSON mode through OpenAI API in this process does not improve the model performance (Table S4†). Details of our implementation and evaluation can be found in ESI.†

### 3.2 Comparison to previous studies

As a smart chemical NER tool, the fine-tuned model learned to recognize cross-referencing tokens and to ignore unwanted chemical entities. This is reflected in the comparison (Table 4) between the fine-tuned model and ChemDataExtractor (version 2.1.0),^53,54^ a toolkit for extracting chemical information mainly from scientific literature. Specifically, the comparison is made for the task of compound name recognition, which evaluates the list of compounds (entities) extracted from a reaction. This list is directly available both from the output of ChemDataExtractor and from the ORD-formatted structured data from the fine-tuned model. While ChemDataExtractor is capable of recognizing many chemical entities, it frequently fails to identify referencing tokens, such as “desired product” or “compound 322” (the “Removal” column). It also captures excess chemical entities, such as “1H” from NMR reports (“the “Addition” column). These errors are at least partially attributable to the distribution shift in how procedures are described in our source text paragraphs. We also compare our fined-tuned model with a NER model based on a pre-trained BERT model, MatSciBERT.^55^ This NER model is trained and evaluated using the same USPTO-ORD-100K dataset and is marginally better than the fine-tuned LLaMA model. Considering the significantly lower training cost of the BERT model compared to fine-tuning the LLaMa model (∼10×), the former may be preferable for pure NER tasks.

**Table 4:** Compound name recognition results of the fine-tuned model, ChemDataExtractor, and the MatSciBert model from the test set of USPTO-ORD-100K. In this task, a set of compound names (entities) is extracted from the unstructured text and is then evaluated against the ground truth

Model	Accurate	Removal	Addition	Alteration	Total
Fine-tuned	94.9%	4.1%	2.2%	1.0%	78 408
ChemDataExtractor	76.1%	16.0%	22.7%	8.0%	
MatSciBert	96.6%	2.2%	2.4%	1.2%	

We further test the fine-tuned model on uniproduct reactions from the ChemRxnExtractor^16^ dataset, a set of 123 records with labeled tokens for compound names. All records from this dataset were collected from individual literature passages. These passages can be considered an out-of-distribution challenge to our fine-tuned model: they tend to be defined by general chemical transformations (*e.g.*, “oxidation of A gave B” or “cyclization of A afforded B”) instead of specific actions in synthesis procedures, chemical amount information is rarely present, and named entities in these passages are frequently represented by externally referencing tokens. As expected, the fine-tuned model performs poorly on this dataset, with an accuracy of 62.6% and a tendency to include unwanted tokens (Table S1†). Such a tendency often results from prioritizing chemical entities above referencing tokens. For example, in “by heating tryptophan methyl ester (9) at 140 °C for 3 h” the token “9” is the correct token to extract, while the fine-tuned model only recognizes “tryptophan methyl ester” which is a chemical entity in a more general sense. These results suggest the ChemRxnExtractor dataset differs significantly from USPTO-ORD-100K, which justifies fine-tuning the base LLaMA-2-7B model for the ChemRxnExtractor dataset. Unfortunately, the small size of the ChemRxnExtractor dataset makes it insufficient for fine-tuning and subsequent evaluation (ESI Section S2†).

### 3.3 Reaction role classification

Reaction role assignments that distinguish reactants, reagents, catalysts, and solvents are sometimes used in downstream tasks such as reaction condition recommendation.^56–58^ The reaction role of a compound is context-dependent, *e.g.*, a chemical can serve as a solvent or a reactant in different reactions, and not explicitly stated in procedure text, so this is also not a pure information extraction task. However, since this implicit information is included in fine-tuning, the fine-tuned model learns the conventions about role assignment in a generalizable way, and the inferred assignment is directly available in the reaction_role field. Since each Compound message is allowed to have only one reaction_role, the reaction role assignment is a standard classification problem. While the ORD data schema has more than 10 types of reaction roles defined to cover a variety of situations, in this dataset only three are used for input compounds (CATALYST, REACTANT, SOLVENT). The prediction of one of these three labels for each compound defines the reaction role classification problem (and corresponding definitions of accuracy) discussed in this section We exclude ProductCompound messages in this section because they always have a reaction_role of PRODUCT in this dataset. We also evaluate a popularity baseline that makes classification decisions based on the role frequency of compounds in the training dataset; roles are uniformly randomly assigned in the case of ties or unseen compounds.

**Fig. 4:**
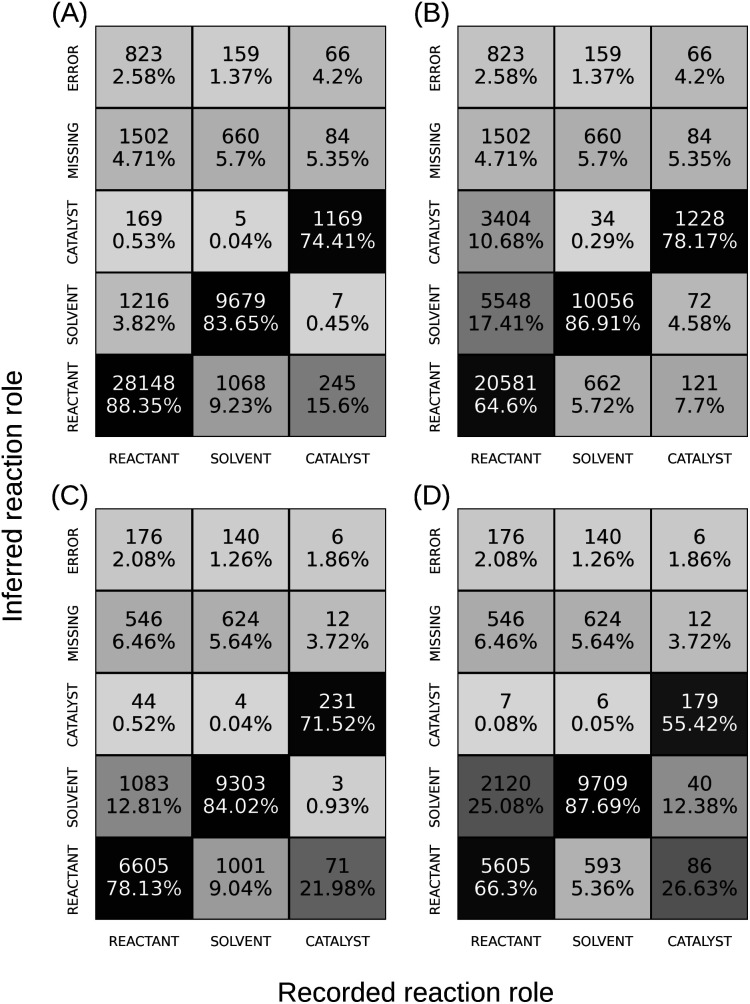
Confusion matrices of reaction role classification for the compounds in the test dataset using (A) the fine-tuned model and (B) the popularity baseline. The results for compounds whose role in the dataset varies from reaction to reaction are shown for (C) the fine-tuned model and (D) the baseline model. Percentage values were normalized using the number of true instances. In addition to three reaction role classes, prediction results can also be labeled as “MISSING” – when the corresponding compound is absent in the extracted ORD record, and “ERROR” – when the name of the extracted compound is incorrect. Note that because the reaction role classification depends on correct extraction of compound names, the first two rows of Fig. 4A and B share identical values. The same applies to the first two rows of Fig. 4C and D.

Fig. 4A shows the confusion matrix of reaction role assignment from the fine-tuned model for all compounds in ReactionInput from the test dataset. The classification accuracy decreases from REACTANT to SOLVENT to CATALYST, with a tendency to mislabel SOLVENT or CATALYST as REACTANT, as expected based on class populations. Compared to extracting compounds of other roles (2.6% for REACTANT, 1.4% for SOLVENT), the model failed more frequently (4.2%) when extracting catalysts. Fig. 4B shows the results from the popularity baseline with similar accuracies for SOLVENT and CATALYST, and lower accuracy for REACTANT compared to the fine-tuned model. A macro-average F1 score of 86.1% is calculated for the fine-tuned model, while the popularity baseline gives 63.5%. For compounds whose reaction role in the dataset varies from reaction to reaction, the difference between the fine-tuned model (Fig. 4C) and the popularity baseline (Fig. 4D) becomes more pronounced: the former exhibits better performance for both REACTANT and CATALYST. These results suggest through fine-tuning the model learned to make role classifications based on reaction context.

## 4 Conclusion

We have demonstrated the application of a fine-tuned LLaMA model for the extraction of structured reaction information from unstructured reaction texts from the USPTO. The fine-tuned model can consistently (99.6%) produce JSON records complying with the highly structured ORD data schema. The fine-tuned model exhibits average accuracies of 91.3% for message level, and 92.3% for field-level extractions. The fine-tuned model can also infer reaction roles that are not explicitly stated in texts, modestly beating the popularity baseline for role classification. While the model may not be accurate enough to be directly used in dataset preparation, it may greatly accelerate information extraction compared to manual extraction, and simplify the job of human curators, especially for detailed, nested data schemas.

As reaction data can include additional non-textual elements, such as reaction schemes and tables for reporting conditions/yields, multi-modality models will be needed to fully organize unstructured data. For reaction schemes, recent developments in the field of optical chemical structure recognition have enabled open-source tools to accurately capture chemical entities from raster images. Notable examples include MolScribe^59^ and RxnScribe^60^ developed by Barzilay and coworkers, as well as ReactionDataExtractor^61,62^ by Wilary and Cole. Table parsing/extraction tools have also been developed for chemistry literature, such as the table parsing module in ChemDataExtractor^54^ and OpticalTable-SQA,^63^ a fine-tuned question-answering language model for table extraction. As multimodal foundation models become increasingly available in fields beyond chemistry, it will be worth exploring their suitability for reaction data extraction.

The obvious use of the fine-tuned model is to support reaction data import to ORD with proper expert validation of the LLM-generated output. For example, as a postprocessing tool to convert unstructured ELN reports to structured data, or a reviewing/proofreading tool to expose as structured data what would otherwise be unsearchable, such as the procedure details buried in supplementary materials of a journal article. Tools presented in this study should contribute to answering the call for standardization in reaction informatics.^1,64^ As aligning reaction text with molecular representation has been demonstrated to be helpful in prediction tasks, the tool developed in this study could also serve as an auxiliary to inform reaction predictive models.^65^

## Data availability

The source code for data processing, the fine-tuning and evaluation scripts, and all fine-tuning/evaluation datasets used in this study can be found at https://github.com/qai222/LLM_organic_synthesis. The fine-tuned model is available at https://doi.org/10.6084/m9.figshare.25485973.

## Author contributions

Qianxiang Ai: conceptualization, data curation, formal analysis, investigation, methodology, software, writing – original draft preparation. Fanwang Meng: data curation, formal analysis. Jiale Shi: conceptualization, methodology, software. Brenden Pelkie: data curation, formal analysis. Connor W. Coley: funding acquisition, supervision, writing – review & editing.

## Conflicts of interest

There are no conflicts of interest to declare.

## Supplementary Material

DD-003-D4DD00091A-s001
