# Supplementary material for: Extracting structured data from organic synthesis procedures using a fine-tuned large language model
Source: Digit Discov. 2024 Jul 31;3(9):1822–31. doi: 10.1039/d4dd00091a (PMC11322921; doi:10.1039/d4dd00091a)
Supplement: DD-003-D4DD00091A-s001 [file DD-003-D4DD00091A-s001.pdf]

Supplementary Information (SI) for Digital Discovery.
This journal is © The Royal Society of Chemistry 2024

Supporting Information for "Extracting Structured Data from Organic Synthesis Procedures Using a Fine-Tuned Large Language Model"

Qianxiang Ai,[a] Fanwang Meng,[a] Jiale Shi,[a] Brenden Pelkie,[b] Connor W. Coley [a*]

1 Sequence length of USPTO reaction records

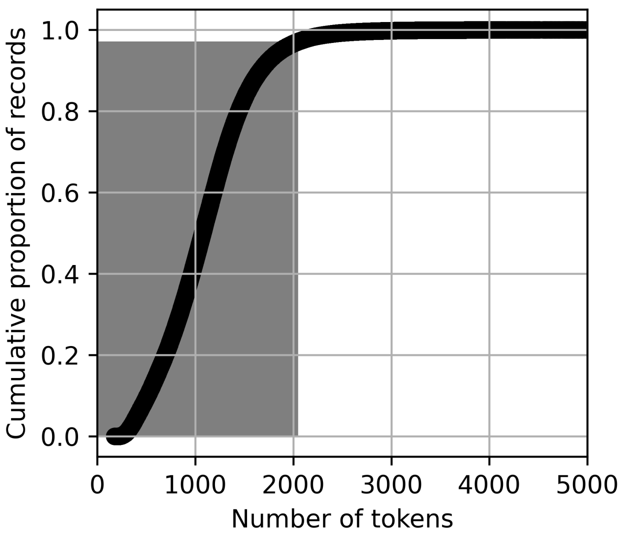

Fig. S1 Cumulative proportion of 1339260 USPTO reaction records as a function of the maximum number of tokens (sequence length limit). The shaded area denotes the 1300613 records within the sequence length limit.

2 Results from the ChemRxnExtracton dataset

Table S1 shows the NER results for two fine-tuned LLaMA-7B models. The first row comes from the model fine-tuned using the training set of USPTO-ORD-100K (the main focus of this manuscript), and it is tested on the entire uniproduct `ChemRxnExtracton` dataset. The second row describes the model fine-tuned using the training set from a random 9:1 train:test split of the `ChemRxnExtracton` dataset, which is tested on the test set from the aforementioned random split. We note while the second fine-tuned model is able to produce valid ORD-formatted JSON, the test set (12 records) is too small to allow meaningful conclusions.

3 Notes on yield extraction and evaluation

In a structured ORD record investigated in this study, a product can have two `ProductMeasurement` messages describing the yield of this product: One for the reported yield which can be found in the procedure text, and the other for the calculated yield which cannot. In our data pipeline, if the integer part of a yield value cannot be found in the procedure text then this `ProductMeasurement` message is dropped out from the record (main text section 2.2 Calculated yield). We choose to only detect the integer part of a yield value to avoid erroneous matching caused by different rounding methods and reporting conventions. This, however, could still lead to situations where the yield values that are not reported in the procedure text remain in the ORD record when all yield values share the same integer part. In the following example (`ord-1f43f796680147a3869d7928c02529ac`),[1] the yield is reported as "89%" in the procedure text.

> A flask was charged with tert-butyl N-[(4aR,11bR)-3,3,11b-trimethyl-10-nitro-4,4-dioxo-5,6-dihydro-4aH-[1]benzothiepino[4,5-b][1,4]thiazin-2-yl]-N-tert-butoxycarbonyl-carbamate (1.18 g, 2.071 mmol), 10 wt.% Pd/C (0.220 g, 0.207 mmol), EtOAc (4.14 ml) and MeOH (4.14 ml). The flask was purged with nitrogen, evacuated, and filled with hydrogen. The reaction was stirred at RT under hydrogen atmosphere for 16 h. The reaction was incomplete, so another 100 mg of 10 wt.% Pd/C was added, and stirring under hydrogen was continued. After 8 h, the reaction was complete. The mixture was filtered through Celite and washed with EtOAc. The filtrate was concentrated to provide tert-butyl N-[(4aR,11bR)-10-amino-3,3,11b-trimethyl-4,4-dioxo-5,6-dihydro-4aH-[1]benzothiepino[4,5-b][1,4]thiazin-2-yl]-N-tert-butoxycarbonyl-carbamate (1.00 g, 89%) as a dark foamy solid.

However, in the structured ORD record, in addition to the percentage yield value of "89.0", another calculated percentage yield of "89.5" is also present.


```
"measurements": [
    {"type": "YIELD",
     "details": "PERCENTYIELD",
     "percentage": {"value": 89.0}},
    {"type": "YIELD",
     "details": "CALCULATEDPERCENTYIELD",
     "percentage": {"value": 89.5}},
     ...
```


Both of the yield values remain in the ORD record after applying our data pipeline. Table S2 shows the field-level evaluation results for reported and calculated yields, where the fine-tuned model can accurately extract reported yields while tends to skip generating calculated yields.

[a] *Department of Chemical Engineering, Massachusetts Institute of Technology, Cambridge, MA, USA.*
[b] *Department of Chemical Engineering, University of Washington, Seattle, WA, USA.*

Table S1 NER results for two fine-tuned models. Note they are evaluated on two different datasets (see above). As the names of different chemical entities can be very similar, we excluded the case of "Alteration" so a name from the text can be captured either successfully ("Accurate") or unsuccessfully ("Removal").

Fine-tuned by	Tested on	Accurate	Removal	Addition	Total
USPTO-ORD-100K	ChemRxnExtractor	201 (62.6 %)	120 (37.4 %)	168 (52.3 %)	321
ChemRxnExtractor (training set)	ChemRxnExtractor (test set)	26 (65 %)	12 (30 %)	14 (35 %)	40

Table S2 Comparison between reported yield and calculated value extractions.

Message type	Field type	Accurate	Removal	Addition	Alteration	Total
`ProductCompound`	Reported Yield	10920 (92.8%)	778 (6.6%)	543 (4.6%)	65 (0.6%)	11763
	Calculated Yield	814 (18.7%)	3273 (75.3%)	810 (18.6%)		4347

4 Fine-tuning prompt template

The following shows the prompt template used in fine-tuning, where `{procedure_text}`, including the curly brackets, is to be replaced with the unstructured procedure text. Note linebreaks are always explicitly denoted as `\n`.

```
Below is a description of an organic reaction. Extra
ct information from it to an ORD JSON record.\n\n###
Procedure:\n{procedure_text}\n\n###ORD JSON:\n
```

5 Chain-of-thought prompting

In this section we detail our implementation of chain-of-thought prompting for structured data extraction.[2] We compose the prompt to have three parts (Figure S2). The first part summarizes the task at a high level, the second part describes the sequential NER/RE steps to construct a generic ORD JSON record, and the third part includes detailed procedures to extract ORD JSON from two example texts. Due to the complicated structures of `ReactionWorkup` and `ReactionConditions`, we excluded these messages in chain-of-thought prompting. This method is tested with 500 reaction procedure texts using OpenAI's `gpt-3.5-turbo-0125`, which was chosen due to its low cost compared to contemporary `GPT-4` models. The temperature is set to zero for consistent outputs. Out of the 500 completions after repairing JSON format, all of them are JSON parsable, but almost half (249) of them do not comply with ORD schema. Most of these violations of ORD schema originate from the misplacement of outcomes as a part of inputs, and can be fixed programmatically. Other violations include unallowed values of `enum` fields, e.g., the allowed types of a `Compound.identifier` do not include "INDEX" which is however extracted in the completion. After further repairing the completions based on ORD schema, 91 (18.2 %) of them are still invalid ORD records.

Evaluation results for the remaining 409 completions are shown in Table S3, from which a reasonable success rate (61.2 %) for extracting `Compound` is observed, along with a poor success rate of 31.3 % for `ProductCompound`, both using the more lenient routine. Similar results (Table S4) are obtained when the JSON mode is turned on through OpenAI API, which promotes the model to generate syntactically valid JSON strings. Note only 351 out of 500 completions generated with JSON mode are valid ORD records. This prompting method is also limited by human-crafted instructions and the context window of the model, and, considering there are more than 600 different fields defined in ORD schema, preparing examples and steps to extract a full Reaction record seems impractical. However, chain-of-thought prompting can still be a low-cost, less-accurate handle for structured data extraction at the compound level when fine-tuning is not available.

6 Supplementary files

All supplementary files can be found at `https://github.com/qai222/LLM_organic_synthesis`. These include:

- The full reaction record in Figure 2 at `https://github.com/qai222/LLM_organic_synthesis/blob/main/manuscript/preprint/fig2_full_record.json`;
- The list of all ORD dataset ids used in our data pipeline at `https://github.com/qai222/LLM_organic_synthesis/blob/main/workplace_data/uspto/dataset_ids.txt`.

7 Numerical error in reaction temperature extraction

While it is possible to use numerical error measure such as the mean squared error for numeric fields, in this study such measure is not used as we prefer the strict evaluation of exact-match accuracy for the information extraction task. A practical reason is, for some fields, errors from addition/removal happen more frequently than alteration. For example, when extracting the temperature fields in `ReactionConditions`, the errors from addition/removal account for 3.2%/2.4%, mostly due to mis-extracting a workup temperature as the reaction condition temperature (or the reverse), and errors from alteration only account for 1.2%. The percentages here are calculated using the method used to produce Table 3 but restricted only to temperature values in `ReactionConditions`. The extracted values, disregarding the

Table S3 Evaluation results at the message level (Evaluation Metric 1) and the leaf field level (Evaluation Metric 2) for completions generated using chain-of-thought prompting on `gpt-3.5-turbo-0125`. The "Path" column denotes the path of the corresponding messages in a Reaction message. The success rates are calculated based on "Accurate" messages/leaf fields. The percentages were calculated using the total number of messages/leaf fields found in ground truth records.
* These values were calculated using a more lenient routine detailed in the main text.

Message type	Path	Accurate	Removal	Addition	Alteration	Total
`Compound`	inputs	955 (52.3 %)	288 (15.8 %)	196 (10.7 %)	584 (32.0 %)	1827
		1118* (61.2 %)			421* (23.0 %)	
`ProductCompound`	outcomes	29 (6.9%)	93 (22.0%)	5 (1.2%)	300 (71.1%)	422
		132* (31.3%)			197* (46.7%)	
**Message type**	**Field type**	**Accurate**	**Removal**	**Addition**	**Alteration**	**Total**
`ProductCompound` & `Compound`	identifiers	3273 (74.8%)	696 (15.9%)	460 (10.5%)	407 (9.3%)	4376
	amount	2570 (80.8%)	567 (17.8%)	427 (13.4%)	45 (1.4%)	3182
	reaction role	1446 (66.0%)	488 (22.3%)	217 (9.9%)	259 (11.8%)	2193

Table S4 Evaluation results for completions generated using chain-of-thought prompting on gpt-3.5-turbo-0125 with JSON mode turned on. See the caption of Table S3 for more details.

Message type	Path	Accurate	Removal	Addition	Alteration	Total
`Compound`	inputs	877 (53.8 %)	376 (23.1 %)	89 (5.5 %)	377 (23.1 %)	1630
		952* (58.4 %)			302* (18.5 %)	
`ProductCompound`	outcomes	35 (9.6%)	68 (18.7%)	7 (1.9%)	261 (71.7%)	364
		164* (45.1%)			132* (36.3%)	
**Message type**	**Field type**	**Accurate**	**Removal**	**Addition**	**Alteration**	**Total**
`ProductCompound` & `Compound`	identifiers	2790 (71.76%)	872 (22.4%)	282 (7.3%)	226 (5.8%)	3888
	amount	2268 (77.83%)	564 (19.4%)	459 (15.8%)	82 (2.8%)	2914
	reaction role	1273 (65.45%)	544 (28.0%)	104 (5.4%)	128 (6.6%)	1945

```
Act as a professional researcher in organic
chemistry. You are asked to extact information
from a given `reaction_text` and export the
information to a ORD JSON record. A JSON record
is an ORD JSON if it uses the Open Reaction
Database (ORD) schema.
```

```
I will describe the 5 steps of this task. I will
then guide you step by step to perform this task
on one examplar `reaction_text`. I will then give
you a new `reaction_text` and you need to
generate the JSON record.

Step 1: Identify all the chemicals in the given
`reaction_text`. An chemical identifier can be
the name of a compound, for example, `methanol`.
An identifier can also be an index or a generic
description, for example, `compound 6`, or
`desired compound`.
Step 2: <CONTINUE TO DEFINE STEPS>
```

```
Here is the first example. `reaction_text` is the
text between two delimiters ```and ```. The
exported ORD JSON record is the text between two
delimiters ### and ###.

`reaction_text` = ```<EXAMPLE REACTION TEXT>```

Here is the workflow how to extract information
from this `reaction_text` and export them to a
ORD JSON record.

Step 1: This reaction involves six chemicals. 4-
aminobenzotriazole, acetic acid, potassium
bromide, ammonium molybdate, hydrogen peroxide,
desired product.
Step 2: <CONTINUE TO DEFINE STEPS>
example_ORD_JSON = ###<EXAMPLE ORD JSON>###

Here is the second example.
<CONTINUE TO THE SECOND EXAMPLE>
```

Fig. S2 An example prompt used for chain-of-thought prompting. The three text chunks correspond to the three semantic parts discussed in Section S5. Texts in angle brackets, including the brackets, are defined by the two examples and are omitted for clarity. The full prompt can be found at `https://github.com/qai222/LLM_organic_synthesis/blob/main/workplace_cot/cot_prefix.txt`.

addition/removal cases, have a mean squared error of 31.95 (a root mean squared error of 5.65) degrees Celsius. The extracted values that are not identical to the ground truth values (i.e. the alteration cases) are shown in Fig. S3.

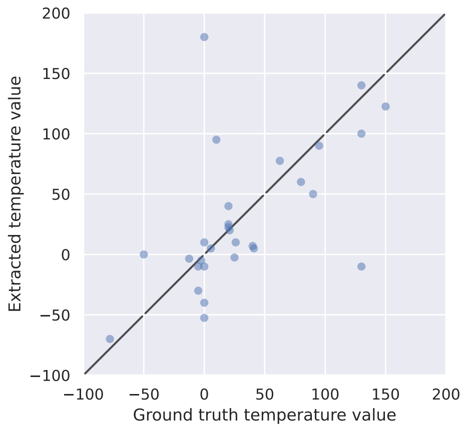

Fig. S3 Temperature values extracted from `ReactionConditions`. Temperature values and units are stored in two separate fields in ORD. All temperature units are Celsius in this dataset. Only the 1.2% of extracted values that are erroneous due to alternation are shown.

Notes and references

1 R. White, J. R. Allen, O. Epstein, F.-T. Hong, Z. Hua, J. B. Human, P. Lopez, P. R. Olivieri, K. Romero, L. Schenkel, J. Stellwagen, N. A. Tamayo and X. M. Zheng, *Fused multi-cyclic sulfone compounds as inhibitors of beta-secretase and methods of use thereof*, 2016, https://patents.google.com/patent/US9309263B2/en?oq=US9309263B2.

2 J. Wei, X. Wang, D. Schuurmans, M. Bosma, B. Ichter, F. Xia, E. Chi, Q. Le and D. Zhou, *Chain-of-Thought Prompting Elicits Reasoning in Large Language Models*, 2023, http://arxiv.org/abs/2201.11903, arXiv:2201.11903 [cs].
